# An assessment of the operationality and factors influencing the effectiveness of rabies surveillance in Gombe State, Nigeria

**DOI:** 10.1371/journal.pntd.0012154

**Published:** 2024-05-07

**Authors:** Adebanwo Kuye, Mishel Dauda, Anthony Oche Ameh, Molta Idris Danladi, Yakubu Joel Atuman, Grace Sabo Nok Kia, Barbara Häsler

**Affiliations:** 1 Department of Pathobiology and Population Sciences, Royal Veterinary College, Hatfield, United Kingdom; 2 Regional Disease Surveillance Enhancement Project (REDISSE), Gombe State Ministry of Agriculture and Animal husbandry, Gombe, Nigeria; 3 Ahmadu Bello University, Zaria, Nigeria; 4 Gombe state Ministry of Agriculture and Animal Husbandry, Gombe, Nigeria; 5 Bauchi Outstation Diagnostic Laboratory, National Veterinary Research Institute, Jos Plateau State, Vom, Nigeria; 6 Department of Veterinary Public Health and Preventive Medicine, Faculty of Veterinary Medicine, Ahmadu Bello University, Zaria, Nigeria; The University of Sydney School of Veterinary Science, AUSTRALIA

## Abstract

Rabies remains a burden in Africa, disproportionately affecting the most vulnerable despite the availability of effective vaccines. Nigeria, the most populous African country, needs rapid disease control actions and commitments to achieve the goal of eliminating dog-mediated rabies by 2030. Surveillance is an essential element of effective disease control strategies. This study examined the current state of operationality of the rabies surveillance system for early case detection and management in Gombe state, Nigeria, through a One Health lens. It further examined the barriers impeding the effectiveness of the surveillance based on the perception of surveillance workers. Qualitative and quantitative methods were used to assess the structure of the system and its functioning. Data on dog bite and rabid cases obtained from the veterinary services in Gombe state were analysed descriptively. A total of 13 key informants were interviewed using a semi-structured interview guide. Qualitative data were analysed using thematic analysis to explore in depth the factors that influenced the operationality of the system. A total of 157 potential human exposures to rabies were identified in this study, out of which two people reportedly died at the health facility after showing symptoms highly suggestive of rabies. In terms of rabies surveillance and control, cross-sectoral collaboration was found between the human health and veterinary sectors for risk assessment of potential rabies exposures and its management. Some identified factors affecting the operations of the surveillance were inadequate funding, lack of infrastructure, lack of feedback from higher authorities and insufficient knowledge of rabies prevention and management. To improve the capacity for case detection and management within the state, the appropriate authorities may focus on increasing awareness about the disease to the populace to increase the number of cases identified by the system, employ more workers and strengthen the surveillance capability of existing workers.

## Author summary

Rabies continues to be a problem in Africa, affecting vulnerable populations, despite effective vaccines being available. Nigeria, being the most populous country in Africa, needs to take prompt action to be able to eliminate dog-mediated rabies by 2030. Surveillance is an essential part of effective rabies control. This study used a mixed-methods approach to investigate how well the rabies surveillance system in Gombe state, Nigeria, is working to detect and manage cases early.

A total of 157 potential human exposures to rabies were found with two deaths suggestive of rabies. The human health and veterinary sectors were found to work together to assess risks and manage potential rabies exposures, but several factors were identified that compromised effectiveness of surveillance. Key barriers were lack of funding, infrastructure, feedback from higher authorities, and knowledge of rabies prevention and management. To improve the system, authorities are encouraged to raise awareness about rabies, employ more workers, and strengthen the capabilities of existing workers in Gombe state. By doing this, they can better detect and manage rabies cases and work towards eliminating the disease.

## Introduction

Rabies, despite being completely preventable via pre and post-exposure vaccination and immunoglobulins still causes estimated annual deaths of 59,000 people mostly within Asia (59.6%) and Africa (36.4%) [1,2]. The lack of accurate records about the true prevalence of rabies in humans and animals within Africa prevents full understanding of its burden and poses significant challenges to current prevention and control efforts [3–5].

Nigeria, the most populous African country remains in the list of countries with dog-mediated endemic rabies in humans that needs to be eliminated [6,7]. Although there is a global drive to eliminate canine-mediated rabies infection in humans by 2030 [8,9], the chances for Nigeria to reach this goal remains a challenge if the status quo remains the same [7]. For instance, the Nigerian government remains burdened by other competing priorities such as inflation, rising insecurity, rising unemployment, brain drain, and religious differences which negatively impact its commitments towards rabies control [10–13].

Surveillance is the continuous collection, analysis and interpretation of health-related data in populations to increase the likelihood of timely detection of health threats and inform disease mitigation actions [14,15]. Rabies surveillance information provides insights on the geographical distribution and epidemiology of animal bites and rabies cases and helps to monitor the effectiveness of rabies mitigation strategies [16,17]. Such insights can be employed in both the design of rabies control strategies and their implementation [18,19]. The One Health (OH) approach advocates for interdisciplinary efforts to address efficiently risks at the human-animal-ecosystem interface [20] and to promote the health of humans, animals and the environment. Collaborative and multi-sectoral efforts have been shown to add value when compared with individual sectors working in silos [21,22]. The OH approach has been identified by the global health security agenda as an integral part to achieve health security against the threat of infectious diseases [23]. Recent analyses from the World Bank suggest that the OH approach when used at country level for disease prevention could yield about $30 billion per year globally as return on investment [23,24].

Rabies surveillance in Nigeria is conducted by both the health and veterinary sectors. The health sector relies on the use of the WHO Regional Integrated Disease Surveillance and Response (IDSR) strategy which constitutes an evidence-based approach employed to strengthen national public health through promotion of rational use of scarce resources and integration of surveillance activities for collection of rabies data in humans [25,26]. Despite the existence of a standard reporting procedure, previous studies indicated that underreporting is common [5,27–29]. The standard reporting process in Nigeria requires rabies to be reported immediately to either the closest health facility or to the veterinary clinic [30]. The health or veterinary facilities handle and submit information as required by the Ministry of Health and Agriculture, respectively. Between 2016 and 2017, the World Health Organization (WHO) reported only 11 fatalities for Nigeria from an estimated population of 202 million people [31].

The veterinary sector uses the National Animal Disease Information and Surveillance (NADIS) to collect rabies surveillance information from various parts of the country including veterinary clinics, veterinary teaching hospitals and the Ministry of Agriculture [25]. A 2020 study conducted in Benue State, Nigeria [5] found that smaller veterinary clinics rarely followed the standard reporting process which contributes to poor data reliability and quality and hinders a full understanding of the prevalence of the disease.

There is a need to understand the surveillance system and factors that affect effective case detection and inform rabies control activities. An understanding of the underlying factors that affect the capacity of the surveillance system to promptly identify and manage cases effectively can help to improve the performance of the system and develop interventions targeted at the affected population [14,32]. Although a good body of literature exists on rabies within Nigeria, to the authors’ knowledge, no specific assessments were published on the rabies surveillance system in humans and animals. Information obtained from evaluation on the capacity of the surveillance would provide practical feedback to stakeholders on areas which can be targeted for improvement of the system to meet the elimination goal. This study therefore aimed to assess the functionality of the rabies surveillance system in Gombe state in Nigeria and to identify the links between surveillance information transmission for early response across the human and animal health sectors. Gombe state, located in the North-Eastern part of Nigeria, is one of Nigeria’s 36 states plus the Federal Capital Territory. This state was selected because of the presence of the World Bank intervention through the Regional Disease Surveillance Systems Enhancement (REDISSE) programme to increase disease surveillance capacity in the animal and human health sectors [33]. Further, there have been numerous informal reports of dog bites and suspected rabies cases, absence of published information on rabies cases and surveillance activities in the state. Also, the heightened insecurity problems intensify the vulnerability of local communities to rabies in the state thereby affecting the choice of intervention strategies employed.

## Methods

### Ethical statement

Ethical approval for the primary data collection was received from the Social Science Research Ethical Review Board, Royal Veterinary College, University of London; approval number URN SR2021-0147. Permission was also sought and obtained from the Ministry of Agriculture in Gombe state with the assistance of the War Against Rabies Foundation (a charity organization assisting the state with ongoing rabies prevention and control activities) before conducting this study. Oral consent was obtained from each of the participants before conducting the online interview sessions.

### Study location

**Fig 1:**
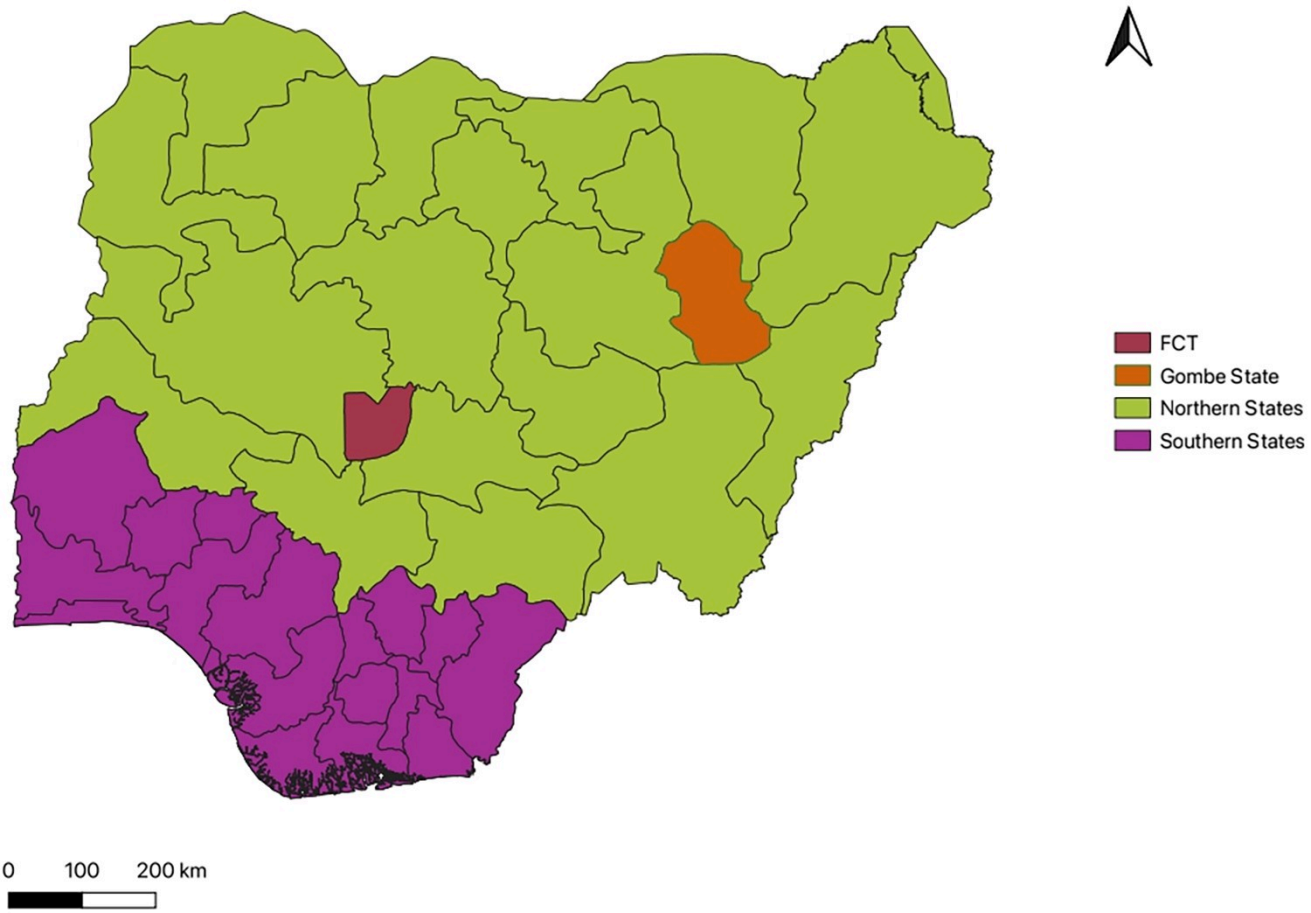
Map of Nigeria highlighting the study location. FCT = Federal Capital Territory. Source of basemap shapefile: https://www.diva-gis.org/gdata.

This study was conducted in Gombe State (10.3638° N, 11.1928° E), located in the North-Eastern part of Nigeria (Fig 1). The state has 11 local government areas with an estimated population of 3 million people who utilize farming as the major source of livelihood [34,35].

### Study design and One Health surveillance conceptualisation

This study utilized a mixed-methods approach for data collection. Secondary dog bite cases and accessible case reports used in the management of dog bite victims within the study location between January 2019 and March 2021 were retrieved from the state Ministry of Agriculture and Animal Husbandry.

Semi-structured interviews were conducted online with key informants within the state and at the federal levels from May to June 2021 to gain insights on the existing disease reporting process and the functionality of the rabies surveillance system. A question guide was used containing demographic questions for respondents and questions on the structure and organisation of the surveillance system (S1 Text).

To inform the development of the interview guide and prepare for the interviews, One Health surveillance for rabies was conceptualised as follows: the collaboration, communication, coordination, and capacity building across sectors to achieve integration of human, animal and/or environment (where relevant) surveillance data with the purpose of informing mitigation actions (e.g., targeted vaccination in humans or rabies control in dogs) [36]. The integration can occur in any of the core elements of surveillance, i.e., data collection and collation (e.g., gather data on rabies cases in both animals and humans to understand the patterns of transmission and the species involved), analysis (e.g., examine the links between animal reservoirs, vectors, and human cases, considering factors such as ecological changes, wildlife behaviour, and human activities; develop algorithms to detect potential outbreaks or increases in rabies cases in animals before they spread to humans), or the use of the resulting information to inform decisions on rabies prevention and control (e.g., joint decision making on strategies that consider animal vaccination, human rabies vaccination, community education, and wildlife management to control rabies effectively).

**Fig 2:**
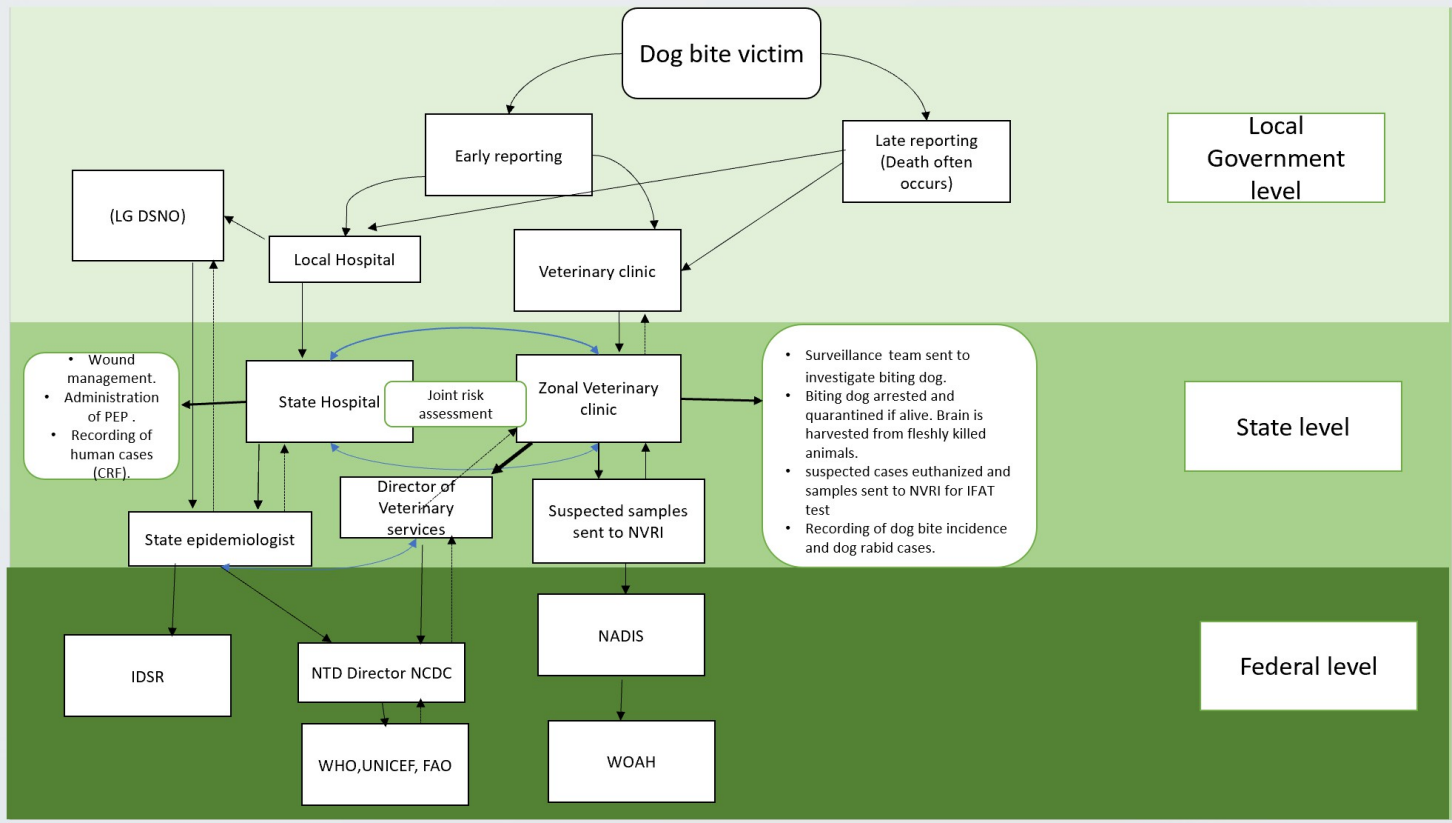
Surveillance wiring diagram of the rabies reporting chain and information flow used for the interviews. Abbreviations: LG DSNO: Local Government Disease Notification and Surveillance Officers; NVRI: National Veterinary Research Institute; NTD: Neglected Tropical Diseases; NCDC: Nigeria Centre for Disease Control; NADIS: National Animals Disease Information and surveillance; WOAH: World Organisation for Animal Health; WHO: World Health Organization; FAO: Food and Agriculture Organization and UNICEF: United Nations International Children’s Emergency Fund.

For the interviews, an initial overview of surveillance was generated in the form of a wiring diagram based on the National Rabies Elimination Guideline and the Nigerian One Health Strategic Plan to illustrate the rabies reporting chain, information flow, and the different stakeholders involved in the reporting process (Fig 2). According to that Guideline, a suspect case is defined as a bite victim showing symptoms such as headache, neck pain, nausea, fever, fear of water, anxiety, agitation, abnormal tingling sensations or pain at the wound site that is yet to be laboratory confirmed. A confirmed rabies case is a suspected case that has been laboratory confirmed. This initial diagram was developed in preparation for the interviews and as a basis for discussion. During the interviews, the interviewees were invited to comment on the diagram, add missing elements or make corrections; this information was used to refine the diagram.

They were also asked questions to assess the operations and effectiveness of the surveillance system including simplicity, acceptability, data collection/storage/management, timeliness and usefulness [37,38] (S1 Text). All interview sessions were conducted online (via zoom) in English by the first author and recorded after oral consent (S2 Text) was obtained from each of the participants. The duration of the interviews with informants ranged from 30 to 70 minutes. Recordings from interviews were transcribed by a professional transcriber into text using Microsoft word.

The key informants recruited for this study were selected purposively as informants act as a rich source of information central to research themes [39] and allow diversity of data [40]. They were selected because they work in or are in close contact with the rabies surveillance system, i.e., are knowledgeable of the system and how it works. The key informants comprised of 2 veterinarians, 1 livestock superintendent, 1 physician, 1 nurse, 2 epidemiologists, 1 veterinary epidemiologist, and 5 public health officials working at different levels of the rabies surveillance system (Table 1).

**Table 1:** Affiliations of key informants.

Profession of key informants	Affiliation
Veterinarian 1	Gombe State, Ministry of Agriculture
Veterinarian 2	Gombe State, Ministry of Agriculture
Physician	Gombe State, Ministry of Health
Nurse	Gombe State, Ministry of Health
Epidemiologist 1	Gombe State, Ministry of Health
Epidemiologist 2	Nigeria Centre for Disease Control
Veterinary Epidemiologist	Gombe State, Ministry of Agriculture
Livestock Superintendent	Gombe State, Ministry of Agriculture
Public Health Official 1	National Veterinary Research Institute, Jos
Public Health Official 2	Nigeria Centre for Disease Control
Public Health Official 3	Federal Ministry of Agriculture
Public Health Official 4	Federal Ministry of Health
Public Health Official 5	Gombe State, Ministry of Agriculture

They were contacted either by email by the first author or through the help of a research assistant who was physically present at the study location.

### Data handling and analysis

#### Quantitative data

The quantitative data on the case reports were obtained in a standardized format (S3 Text). They were entered manually into a Microsoft Excel (Office 365) sheet. The data set was cleaned, i.e., duplicates and incomplete records were removed, words with different spelling aligned, and the data obtained was filtered using the sort and filter function of Excel to generate summary statistics.

A Pearson Chi-square analysis was performed to test for association between the age of bite victims and the site of the bite. The bite victims were classified into four age categories (0<16 years; 16–30 years; >30 years and unknown). A p-value of less than or equal to 0.05 was considered to be significant. The Pearson Chi-square analysis was conducted using statistical Packages for Social Sciences (SPSS) version 25.

#### Qualitative data

Thematic analysis as described by Braun and Clarke [41] was utilised in analysing the qualitative data. First, familiarisation of the data was achieved through reading the transcripts several times, after which they were coded. Data coding was performed using Nvivo software (QSR International). Coding as defined by Sutton and Auston [42], i.e., the process of identifying the similarities and differences across an entire data set, was pursued in an inductive way. Themes were formed by synthesizing codes from the transcripts, enabling understanding of the surveillance system and identifying opportunities for its improvement.

## Results

### Key informants’ demographics

The majority of the key informants interviewed had more than five years of experience working on rabies surveillance (Table 2). The educational qualification of informants ranged from a diploma to a doctoral degree.

**Table 2:** Demographics of key informants.

Variable		N = 13 (100%)
**Years of experience in surveillance**	0 < 2	1 (8%)
	2–5	1 (8%)
	> 5	11 (84%)
**Gender**	Male	10 (77%)
	Female	3 (23%)
**Educational qualification**	Diploma	2 (15%)
	Bachelors	3 (23%)
	Masters	7 (54%)
	Doctoral	1 (8%)
**Surveillance training**	Received training	6 (46%)
	No training received	7 (54%)

### Description and operation of the rabies surveillance system in Gombe state

**Fig 3:**
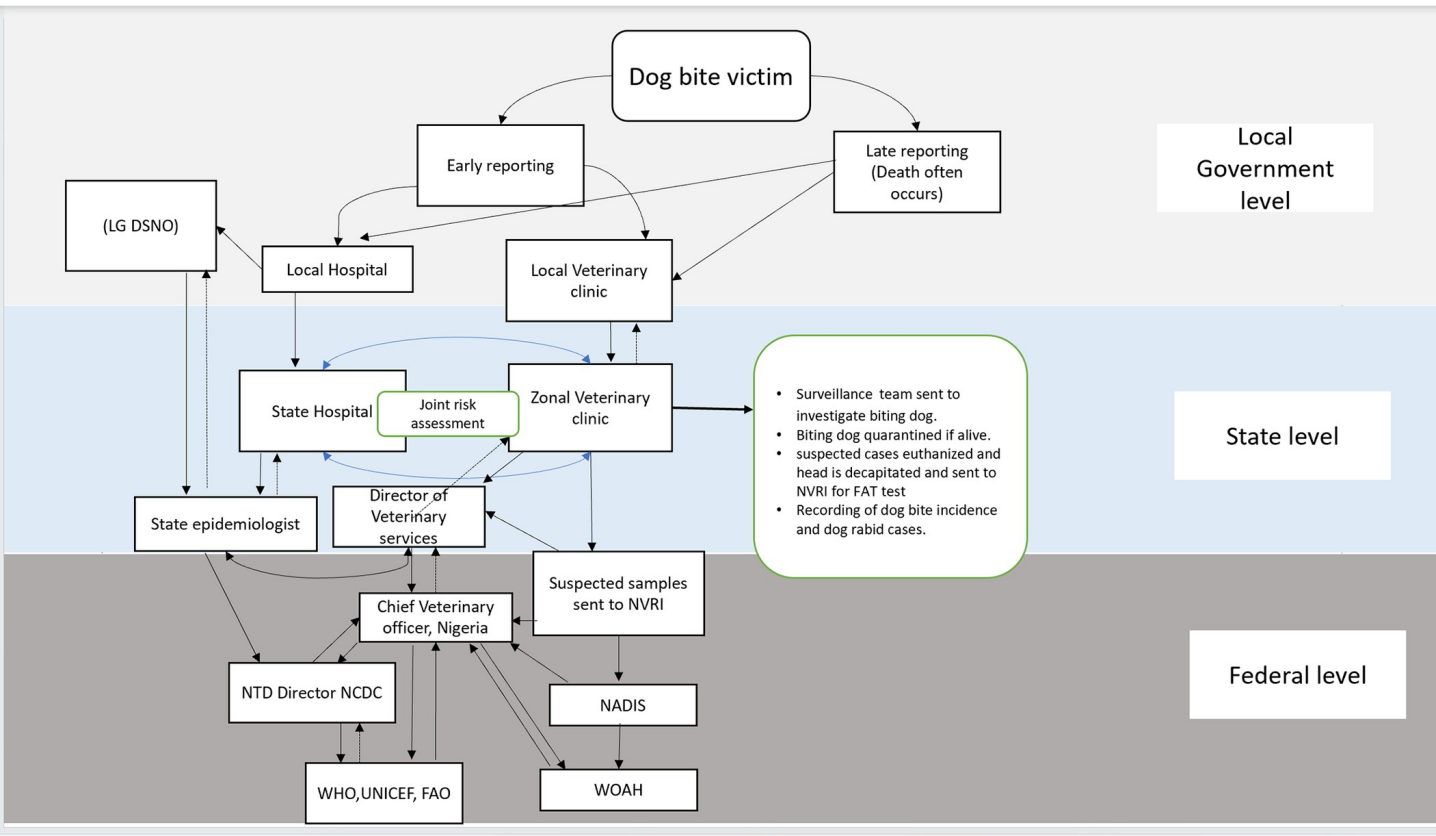
Graphical overview of information flow and disease reporting structure of the surveillance system for rabies detection in Gombe State. Abbreviations: LG DSNO: Local Government Disease Notification and Surveillance Officers; NVRI: National Veterinary Research Institute; NTD: Neglected Tropical Diseases; NCDC: Nigeria Centre for Disease Control; NADIS: National Animals Disease Information and surveillance; WOAH: World Organisation for Animal Health; WHO: World Health Organization; FAO: Food and Agriculture Organization and UNICEF: United Nations International Children’s Emergency Fund.

Fig 3 shows a graphical overview of how the rabies surveillance system in Gombe state works based on the initial wiring diagram created (Fig 2) and the inputs received from the key informants. The inputs received from the interviews which differentiate Fig 3 from Fig 2 is the State epidemiologist reporting to the NTD Director of the NCDC instead of reporting to the IDSR and the role of the Chief Veterinary Officer in the surveillance system at the National level who shares and receives information from the NTD Director of the NCDC, WHO, UNICEF, FAO and WOAH.

The surveillance activities for rabies are organized separately by the health and veterinary sectors, with some areas of integration. The surveillance system is passive and relies both on the victim’s ability to report cases of dog bites and symptoms highly suggestive of rabies to the nearest health or veterinary facility and the ability of the health personnel to report such cases to the appropriate authority. Following a dog bite case, the victim–if well informed about rabies–reports to either the veterinary or health personnel in the local government. The health officials in turn refer the victim to the state capital for a joint risk assessment by both the health and veterinary department. The risk assessment is based on the specified outline in the National Rabies elimination guidelines, 2016 [30]. This involves determining the likelihood of exposure of the victim to rabies by using information such as the pre-exposure vaccine status of the victim, vaccination status of the biting dog, clinical signs displayed by the biting dog during quarantine, and laboratory results of the biting dog (as explained below). The surveillance team members are often sent from the veterinary department to the location where the dog bite case occurred to identify the biting animal and other dog bite victims. The dog, if found, is quarantined for ten days by the owner under veterinary supervision whenever the vaccination record of the dog is unverifiable. Sometimes, the biting dog remains unidentified by the community; other times the biting dog is killed by the community prior to the arrival of the surveillance team. The head of the killed dog is decapitated on the arrival of the surveillance team and sent to the central laboratory, National Veterinary Research Institute (NVRI), Vom, Nigeria. Similarly, during quarantine, dogs showing clinical signs of rabies are euthanized, and the brain samples are also sent to NVRI for Fluorescent Antibody Test (FAT) for confirmatory diagnosis.

On the other hand, the bite victim presented to the health facility is examined and post-exposure prophylaxis (PEP) is initiated. PEP consists of administration of tetanus shots and the first shot of rabies post-exposure vaccination. A dog bite victim requires at least five doses of human rabies vaccine for effective PEP which costs ₦ 22,500 (US $29) in Gombe State. Due to the high cost of the rabies vaccine, the administration of the remaining dose of the post-exposure vaccination is discontinued for victims if the biting dog shows no clinical sign of rabies after ten days in quarantine or if the rabies test result of the biting animal returns negative. Post-exposure vaccinations are continued for a confirmed case based on the rabies case definition. A confirmed rabies case is a laboratory diagnosed case of a person showing symptom of headache, neck pain, nausea, fever, fear of water, anxiety, agitation, abnormal tingling sensations or pain at the wound site and who had contact with a rabid animal. Rabies immunoglobulin (RIG) use is limited in Gombe State because of its limited availability. RIG costs ₦ 60,000 (US $79). The full dose of RIG is divided into two parts, the first part is infiltrated into the wound site and the other part is given intramuscularly to the victim.

### Dog bite, human and dog rabies cases in Gombe state between January 2019-March 2021

Based on the available records from the veterinary department, a total of 157 people visited either the veterinary department or hospitals to report the dog bite cases whether or not they could afford the cost of the PEP treatment. Once the bite victim identifies the dog, the dog is arrested by the veterinary department to do a risk assessment on the dog. Once the results of the investigation are completed, the health department is briefed to determine the next line of treatment for the bite victim. Out of the 157 victims, 105 (67%) were male and 52 (33%) were female. A total of 83/157 (53%) were children less than 16 years of age, and 72/157 (46%) were adults (Table 3). The most frequent body location for bites was the leg with 92/157 (59%) bite cases, followed by the hand with 47/157 (30%) bite cases. Gombe local government had the highest incidence of 123/157 (78%) dog bites cases. Among all the cases of dog bites, two fatalities were recorded after these individuals showed symptoms which were highly suggestive of rabies on presentation to the health facility. The first victim was a 7-year-old boy presented to the State Veterinary Clinic, Gombe showing symptoms of hydrophobia, excitation, partial paralysis of the lower limbs, dilation of the pupils and hallucination. History obtained from the victim’s family indicated that a stray dog had bitten the boy. The bite was unprovoked as the rampaging dog had invaded the compound of the boy’s home. The victim died the next day at the Federal Teaching Hospital. The other victim was a 12-year-old boy brought into State Veterinary Clinic, Gombe. He showed nervous signs highly suggestive of rabies. The boy was reported to have been bitten over a month ago on his right hand. Traditional medicine was used for the boy by his family after the incident of the dog bite. The victim died the next day at the hospital after treatment was initiated.

**Table 3:** Demographics of human victims of dog bites and vaccination status of biting dogs.

Variable		N = 157 (100%)
**Age range of victims (years)**	0 < 16	83 (53%)
	16–30	33 (21%)
	> 30	39 (25%)
	Unknown	2 (1%)
**Gender of victims**	Male	105 (67%)
	Female	52 (33%)
**Vaccination status of biting dogs**	Vaccinated	12 (8%)
	Unvaccinated	145 (92%)
**Site of bite**	Body	12 (8%)
	Face	5 (3%)
	Hand	47 (30%)
	Leg	92 (59%)
	Unknown bite site	1 (1%)

Four of the 157 (3%) biting dogs were tested for rabies by direct FAT at the central laboratory; 3/4 (75%) dogs tested positive. A total of 145/157 (92%) of the biting dogs were classified as unvaccinated; this included the unidentified dogs whose vaccination status could not be verified (Table 3).

The Chi-square test showed that there was significant association (χ2 = 247.84; P = 0.000) between the age of the victim and the body part that was bitten (Table 4).

**Table 4:** Relationship between the age of the bite victims and the bite site.

Age (years)	Site of bite					Total
	Body	Face	Hand	Leg	Unknown	
0<16	0 (0.0%)	0 (0.0%)	0 (0.0%)	83 (52.9%)	0 (0.0%)	83 (52.9%)
16–30	0 (0.0%)	0 (0.0%)	24 (15.3%)	9 (5.7%)	0 (0.0%)	33 (21.0%)
>30	12 (7.6%)	4 (2.5%)	23 (14.6%)	0 (0.0%)	0 (0.0%)	39 (24.8%)
Unknown	0 (0.0%)	1 (0.6%)	0 (0.0%)	0 (0.0%)	1 (0.6%)	2 (1.3%)
Total	12 (7.6%)	5 (3.2%)	47 (29.9%)	92 (58.6%)	1 (0.6%)	157 (100.0%)

(Chi-square (χ2) = 247.84; df = 12; P-value = 0.000)

### Thematic analysis: Factors influencing the functionality and performance of the system

#### Various barriers exist to effective rabies surveillance and control

Informants identified different barriers to surveillance which hindered the effective control of rabies. Inadequate knowledge of rabies, especially in rural communities coupled with traditional health-seeking behaviours and beliefs, was identified as a significant obstacle. It affected people’s ability to seek appropriate medical care from health professionals.

“*In most of our local governments*, *they don’t know the clinical signs … of rabies*. *They take rabies incidence as if it is a diabolic thing*, *they may say “it is devil*… *There was a case that happened early this year in one of the local governments where a boy was bitten by a rabies infected dog*. *It took so long before the case was reported to the area veterinary officer*. *The boy started showing clinical signs of rabies and before it came to our notice*, *the person died*. *These issues are as a result of lack of education and awareness of what the rabies disease is all about”- Participant 003*.

In addition, traditional healers were also described to dissuade victims of dog bites from seeking adequate care from the health facilities. In turn, this was linked to factors affecting the ability of the surveillance system to detect cases in remote areas.

“*Traditional healers*, *they discourage the victims*, *but if they see us they will keep quiet but when we leave there they will start discouraging them that this one is simple o*, *don’t waste your time to go to the hospital*, *so sometimes they are really giving us headache but if they see us they don’t talk*, *they only talk when we are not there”–Participant 004*

Another barrier to effective surveillance was the absence of adequate quarantine facilities to isolate biting dogs for rabies within the state. Informants in both sectors explained that based on the current state of veterinary operations in the Gombe state, efficient quarantine of dogs was dependent on the cooperation of dog owners to efficiently isolate their dogs based on their understanding of the risks involved if a rabid dog was let loose to the community.

“*We don’t keep animals that are suspected with rabies in our clinic because in our clinic we don’t have enough space*. *But most of the time what we do is that we go to the owner’s house and quarantine the dog in its cage and lock the cage and advise him on what to do for the 10 days of observation*. *So that’s what we do”-Participant 004*

Likewise, the sub-optimal vaccination of dogs by their owners due to the cost of vaccination coupled with the movement of unvaccinated dogs from neighbouring states into Gombe was described as another challenge. Informants in both sectors also expressed concerns over the neglect of rabies by the government. They emphasized the need to allocate limited resources to the high-risk areas to optimize efforts used in rabies control.

“*Rabies has been side-lined*… *the government hardly supports cases of rabies*. *The government hardly provides human vaccines when we have cases”-Participant 001*

“*Where you think the problem is*, *that is where you are going to channel all your resources especially because we don’t have adequate resources”-Participant 002*

#### Surveillance needs better resourcing and capacity improvements

Insufficient funding for surveillance activities and inadequate remuneration of staff was described to affect greatly the quality of surveillance. Despite their willingness to continue surveillance activities for rabies case detection, participants expressed frustrations about understaffing, insufficient remuneration and incentives, lack of logistics, and deficient infrastructures to encourage surveillance activities. These problems were mentioned repeatedly by informants at both higher and lower levels of the surveillance system and were described to affect the quality of surveillance information.

“*Our staff are willing to work but most of the time they will say they need transport*, *they need this*, *they need that and sometimes they need mobility to transport themselves to remote areas*. *We don’t have a ride to carry them to the sites and so on and so forth*. *So the serious challenge is lack of logistics or motivation or incentives”- Participant 004*

“*Only 8 veterinarians and three of those veterinarians are at the directorate level at the ministry*. *So*, *we only have five veterinarians left to cater for the needs of eleven local governments*. *It’s actually difficult”-Participant 001*

Equally, several informants stressed the need to retrain, raise awareness, and disseminate up-to-date information to personnel across all levels of the surveillance system to increase their competence in managing and reporting both dog bites and rabies cases. Only 6 (46%) participants explained they had received any training on surveillance.

“*Not everyone understands the case definition for rabies and that’s why there is need for consistent training of disease surveillance agents and then our technical officers in the field”–Participant 001*

A recurring observation among interviewees during the interviews was limited knowledge about the whole operation of the surveillance system, and surveillance activities occurring outside interviewees delineated work responsibility.

“*That is what I said; we don’t have all those information here*. *If you want it*, *you can go through the intermediary; I cannot deploy something that I don’t know”-Participant 006*

#### Motivation, community engagement and data quality influence information generation and flow

The passive surveillance was described to be dependent on the victim’s ability to promptly report cases to the health authorities and health authorities’ promptness in forwarding the case to the appropriate unit. Some informants described the readiness and enthusiasm of some other communities to report cases promptly to the authorities, especially after sensitization programs were done while a few others identified instances of the use of law enforcement agents to ensure case reporting was done.

“*The members of the populace also help in the system*, *they do inform our staff that are within their locality of everything happening to a dog and those that exhibit abnormal behaviour*. *So*, *whatever they see as abnormal to them*, *they quickly inform our staff that are with them in their localities because we do have veterinary assistants in all the wards located closer to the people there*, *so they do assist them*. *And even the community leaders too*, *they have all our contacts and whatever they see as abnormal regarding animals and especially dogs*, *they will tell us directly”- Participant 004*

Most of the informants opined that the motivation of staff was a helpful factor in improving information generated by the system. All informants noted the workers were highly active in ensuring rapid case detection and effective management.

“*We work hand in hand with the state and we have a very good support by the state*. *We have a system in place organized*, *anytime we say there is a rabies case we swing into action even if it is a hard- to- reach area we must find a way to access that place”–Participant 012*

Notably, informants in the health sector acknowledged inefficiencies in the data generation process at their unit and explained it affected the quality of data generated for the surveillance system.

*“There is a weakness in the system… . We miss a lot of data, it’s not all cases of dog bites that are recorded”-* Participant 002

“*Concerning the data*, *actually rabies is rare*, *for now we don’t have a conclusive data for that*…*our data is not too good because we don’t report it”- Participant 010*

## Discussion

An assessment of the rabies surveillance system in Gombe State by this study revealed that intersectoral collaboration was actively employed for joint risk assessment for the management of potential rabies exposure cases. This approach of integrative efforts by different sectors has been shown by others [21,22] to provide added value by preventing discrepancies in the number of cases reported, improving case management and increasing worker’s acceptability of the system as compared to when each sector works independently. Several barriers were also identified that undermines the system’s functionality and effectiveness. The major impediments include insufficient funding, inadequate staffing, inadequate training of personnel, lack of infrastructural resources and mobility for surveillance. These challenges mirror those reported for other neglected tropical disease surveillance systems across Africa, in which rabies is a part of [43,44]. These problems might also contribute to possible reasons for the limited effectiveness of surveillance systems in many African countries [45].

A total of 157 potential human exposure to rabies were identified in this study, out of which two people reportedly died at the health facility after showing symptoms highly suggestive of rabies. Furthermore, children ranked highest as victims of dog bites; the victims that died in this study were also children. This finding is similar to other studies conducted in Nigeria [29,46]. This might be attributed to the inability of children to understand hazardous situations that can trigger a bite event and defend themselves in such situations [28,46]. Likewise, children not understanding the risk of dog bites might fail to report such incidents to their parents preventing immediate medical care [13,28]. Hence, there is need for awareness raising among children and parents to enable them to speak out and for parents to seek medical treatments. Testing of biting animals is critical to determine the course of treatment of bite victims and to ensure mass vaccinations of dogs in the vicinity where the bite occurred. Remarkably, in this study, the testing of biting animals for rabies was found to be exceptionally low. The small proportion tested might be attributed to the insufficient workforce to follow-up on quarantined dogs in victim’s houses or the cost associated with transporting the samples to the central laboratory (NVRI). The IFAT testing is done both at the Nigerian Veterinary Research Institute (NVRI) and at the Ahmadu Bello University (ABU), Zaria. While testing at NVRI is often done for routine Rabies diagnosis, IFAT tests conducted at ABU, is often conducted for research purposes [29]. Moreover, NVRI is closer to Gombe state compared with ABU for rabies testing. Consequently, it might be necessary to employ more veterinary personnel and provide resources to transport these samples or equip smaller laboratories within the state with test kits to conduct other alternative tests recommended by the WOAH, like the direct rapid immunohistochemical test (dRIT) [47]. A study by Eze and colleagues [48] evaluating the economic feasibility of rabies diagnostic tests also suggested using dRIT in resource-limited settings due to its cost-effectiveness compared with the FAT test used in Gombe state.

The proportion of vaccination among the biting dogs reported in this study was also low; this might be due to insufficient knowledge about the importance of dog vaccinations or inadequate knowledge of where to find the vaccines or the veterinary clinics where they are administered [49]. The low dog vaccination coverage is relatable with other studies conducted in Nigeria, where the vaccine coverage has been reported to be below the recommended 70% coverage level for effective rabies control [28,46,50].

The Global Alliance for Rabies Control (GARC) estimates dog vaccination coverage in Nigeria as 12.29% [51]. Some studies conducted in Nigeria indicate 64% vaccination coverage in Bauchi metropolis [52], a coverage of 41% in urban Kaduna and 9% in rural Kaduna [53]. In this situation where rabies is endemic, and only a few dogs are vaccinated, the risk for human exposure to rabies is exceptionally high. Although, free rabies vaccinations are often provided to dogs to commemorate the world rabies day in many states in Nigeria [54], these programmes are often conducted in urban settings rather than rural settings where dog owners cannot afford the cost of vaccination. Lack of awareness about rabies case reporting in rural communities was described as a factor affecting case detection through the passive surveillance system, as cases remain unreported to the health authorities. Traditional approaches were described to be used regularly by victims after dog bite cases. This might be attributed to lack of knowledge on rabies case management. Traditional approaches often involve slaughtering of the dog, and the use of its part for traditional therapy by local healers. This approach disregards the outlined method for suspected rabies management and poses a threat towards the emergence of a new infection towards the handlers of the dogs tissue [55]. Previous studies in Nigeria have isolated the lyssavirus from dogs in trading markets and illustrated that slaughtering, handling and processing dogs provides avenue for exposure of contact with fluids and nervous tissues of dogs which could transmit the disease if the dog is infected [4,28,55]. Community engagement programmes providing information on rabies, targeting both the peri-urban and rural communities, is essential for increasing knowledge and awareness [56]. Although awareness is promoted annually on the World Rabies Day in the form of outdoor sporting events, mass media and vaccination campaigns [27], these events often do not reach remote areas where the most vulnerable populations at high risk of developing rabies can be found. There is a need to scale-up rabies awareness from one-day annual events to ongoing year-round events with coverage of high-risk populations. Heightened awareness of rabies in rural communities can reduce underreporting and increase the desirability of dog vaccinations since community members have become much more knowledgeable about the disease. For example, a community-based surveillance trial conducted in Kenya demonstrated that community engagement and awareness resulted in a higher rate of case detection by the system [57]. However, another study conducted in Peru indicated that having knowledge on the need to seek health care after potential exposures to rabies did not necessarily translate into any action taken by the victims [58]. Thus, in addition to awareness of the community, decision makers need to consider options that can incentivise the desirable behaviours. For example, providing subsidized or free treatment options for dog bite victims and dog vaccination would likely be helpful, as the inadequate financial capacity of the Nigerian populace might deter the expected progress of increased case detection gained through community engagement programs if 40% of the population still lives below $1.1 per day and find it financially demanding to seek appropriate healthcare [59].

The passive surveillance system adopted in Gombe state as reported in this study aligns with the results from a survey of the surveillance systems in African countries by Taylor and Knopf [60] who reported rabies surveillance to be ineffective in 16 out of the 23 participating countries due to the use of a passive surveillance system. To address the challenges associated with this passive surveillance, the use of the One Health surveillance approach needs to be strengthened with inter-sectoral collaboration in data collection, analysis and management to inform decision making. This is expected to translate to improved overall efficiency of case detection and management and the generation of co-benefits. For instance, One Health surveillance has been previously implemented in Kyrgyzstan where joint surveillance in livestock and humans formed the basis for the development of intersectoral and cost-effective control [61]. A similar joint surveillance approach has also been reported in Mongolia, the Philippines, and Tanzania [61–63].

The Integrated Bite Case Management (IBCM), known to have increased rabies case detection in the Philippines, Tanzania and Chad [47,62,63], is a One Health approach for rabies mitigation that links both the veterinary and health sectors to assess the risk of rabies among dog bite victims and the implicated biting animal to ensure optimal treatment. The risk assessment of the IBCM is dependent upon the patients’ ability to present themselves to a health facility following a bite incidence [62]. In a setting like Nigeria where resources targeted at surveillance activities is limited, a potential solution to improve case detection might be to leverage on a free short text messaging system to the central bodies, the Nigerian Centre for Disease Control (NCDC) and the Federal Veterinary Department by dog bite victims or local leaders if bite victims do not have phones. This system could work like this: Once a bite victim sends a text message after a bite incident, the victim receives an auto-generated response in the form of a rabies advisory information on quick actions that can be taken by the victim. It also automatically alerts the veterinary and/or health sector closest to the location of the bite to do a follow-up which will ensure swifter case handling. Such a system could help to increase the number of cases reported and generate a greater understanding of the geographical distribution of bite cases which in turn would inform decisions to conduct mass vaccination and awareness thereby leading to greater overall efficiency and effectiveness of the surveillance system. For this quick messaging system to work, there might be need for incentivizing individuals who successfully use the system to ensure wider acceptability and a need for prompt action by health authorities to ensure confidence of the public in the system. Equally, training of health and veterinary workers in the use of smart phones would be needed to enable the successful use of the technology. Local government health and veterinary sectors collaboration is needed for efficient case handling. Successful real-time information sharing and intersectoral collaboration require political support, funding commitment, and policy implementation for One Health surveillance to enable coordinated case reporting and maximize benefits in rabies surveillance.

The interviews with key informants allowed developing understanding of the current state of operations and the functionality of the surveillance system. Despite the insights gained, this study has a number of limitations. Firstly, interviews with key informants were conducted via Zoom calls. This approach might have limited informant’s ability to speak freely compared with if the interviews were conducted physically; some interviewees were observed to be apprehensive about talking around certain issues regarding the surveillance system. However, there is a possibility that other participants might have also felt more comfortable expressing themselves since their cameras were switched off during the calls and their faces could not be seen. Secondly, informants might have given biased responses due to the need to portray themselves as being socially desirable in the performance of their duties. The findings from this study were based on one out of six geopolitical regions in Nigeria. Further studies would be needed to explore the functionality and operation of other states within the remaining five geopolitical regions in Nigeria to provide a broader perspective on the strength and weakness of the overall rabies surveillance system in the country.

## Conclusion

The outcome of this study offers comprehensive insights into the current operations and state of the functionality of the rabies surveillance system in Gombe state, Nigeria. This research contributes to researchers’ and stakeholders’ knowledge about areas within the surveillance system which can be targeted for reinforcement to improve the system’s overall functionality in line with the Rabies elimination goal by 2030. Through the findings from this study, it is concluded that the overall functionality of the rabies surveillance system in Gombe state can be improved by strengthening the surveillance capacity of workers through retraining of existing staff, employing more veterinarians, increasing the sensitization on rabies within the populace, providing adequate funding and infrastructure while simultaneously leveraging on the existing enthusiasm of workers and collaborative efforts existing at some levels within the system.

## Supporting information

S1 Text Interview questionnaire. (PDF)

S2 Text Information and consent form. (PDF)

S3 Text Gombe State dog bite reporting form. (PDF)
