# Supplementary material for: An assessment of the operationality and factors influencing the effectiveness of rabies surveillance in Gombe State, Nigeria
Source: PLoS Negl Trop Dis. 2024 May 7;18(5):e0012154. doi: 10.1371/journal.pntd.0012154 (PMC11108123; doi:10.1371/journal.pntd.0012154)
Supplement: S1 Text — (PDF) [file pntd.0012154.s001.pdf]

Mapping and questionnaire for interviews

Questionnaire used for conducting interviews in this study:

Demographics

Local government name:

- Akko ☐
- Balanga ☐
- Billiri ☐
- Dukku ☐
- Funakaye ☐
- Yalmatu/Deba ☐
- Gombe ☐
- Kaltungo ☐
- Kwami ☐
- Nafada ☐
- Shongom ☐

Name of the establishment you work in:

Gender: Male ☐ Female ☐

What is the highest level of formal education attained?

- Secondary Education ☐
- Bachelor's Education ☐
- Masters Education ☐
- Doctoral Education ☐

How long have you been working in the rabies surveillance system?

- Less than 2 years ☐
- Between 2- 5 years ☐
- Greater than 5 years ☐

What role do you have within the rabies surveillance system?

Health care worker ☐

Animal Health worker ☐

Surveillance officer ☐

Epidemiologist ☐

Other ☐

Have you received any special training on rabies surveillance? Yes ☐ No ☐

If yes, please describe the training received:

Mapping guide for surveillance system activities

I will show you a map that I have created in Microsoft PowerPoint. The information I used to produce this overview map included the National Rabies Elimination Guideline and the Nigerian One Health Strategic Plan. The map illustrates the rabies reporting channels and information flows of the system. I will ask you a few questions in relation to this map.

1. Is this map an accurate representation of the rabies surveillance system as you know it? Why?
2. Is there anything that is wrongly displayed on this map? Could you describe it?
3. Is there any missing information on the map? If yes, please can you kindly explain them.
4. Based on your knowledge, can you explain the responsibility of your unit as displayed in the map?
5. What data is generated from your unit with regards rabies surveillance?
6. What is done with the data once they are generated? (sent immediately to the next unit or analysed).
7. Based on your knowledge, who else do you most closely collaborate with and what is their role?
8. Can you describe integration mechanisms with your unit and other parts of the system? Integration can be any form of collaboration, sharing, exchange, or coordination in the surveillance system and can refer to data collection, data analysis, use of the information, decision made on the information, governance, budget, or similar. I would be particularly interested in any integration points or mechanisms between human surveillance and animal surveillance?
9. Can you describe areas of inter-operation between human surveillance and animal surveillance? Inter-operation means the ability of the system or components to work with another without any special effort. For example, sharing of a common database between the health and veterinary sector.

Surveillance system evaluation

Different guidelines have been developed for evaluating attributes of the surveillance system attributes. The questions for this interview guide were adapted from existing guidelines such as the CDC, RiskSurv/SurvTools, and SERVAL guidelines.

The questions below refer to the overall surveillance system as shown in the map. If you would like to comment on particular aspects of the system, please specify them.

Simplicity:

This refers to the ease of operations and data flow within the surveillance system.

1. On a scale from 1 to 5 where 1 is simple and 5 is complicated, how would you score the simplicity of the surveillance system spanning both humans and animals? (Is the disease reporting and data management process easy and clear to you?) What is your reason for giving this score?
2. Are there any the objectives of the rabies surveillance system? If yes, what are the objectives?
3. Do you have a case definition for rabies (humans/animals) in your surveillance system? Is it written?
4. How easy is it for you to understand the case definition?
5. In your view, on a scale of 1 to 5, where 1 means extremely simple and 5 stands for complication. How easily does information flow through the surveillance system within one sector? What is your reason for giving this score?
6. On a scale of 1 to 5, where 1 means extremely simple and 5 stands for complication. How easily does information flow *between* Health and veterinary services? What is your reason for giving this score?

Acceptability:

This refers to the willingness of people to actively participate in the surveillance system activities.

1. On a scale of 1 to 5, where 1 means not active and 5 means extremely active. How actively do owners of dogs with suspected rabies or their families or communities report cases to the health or veterinary service? What is your reason for giving this score?
2. On a scale of 1 to 5, where 1 means not engaged while 5 means extremely engaged. How engaged are Doctors/Veterinarians/surveillance officers involved in the active identification of suspected and confirmed rabies cases in humans/animals? What is your reason for giving this score?
3. On a scale of 1 to 5, where 1 means not engaged while 5 means extremely engaged. How engaged are Doctors/Veterinarians/surveillance officers in the active identification of suspected and confirmed humans/animals rabies cases? What is your reason for giving this score?
4. In your view, are there certain groups that you would like to be more active in the surveillance system? Which ones and why?
5. What are the challenges affecting the willingness/motivation of workers within the surveillance system to actively participate in the system?
6. On a scale of 1 to 5, where 1 means zero acceptance and 5 means high acceptability. What is the acceptability of the surveillance information are the veterinary and health sector about

surveillance information generated from each other? What is your reason for giving this score?

Data collection, storage and management:

The use of appropriate data collection and storage methods.

1. Are there any protocols for data collection in your unit? If yes, can you explain?
2. How do you ensure the data is adequately collected by your unit?
3. How and where is the data collected by your unit stored?
4. Is the data stored in a way that allows easy interrogation and analysis? Why?
5. Data quality checks?
6. Is there a document providing a summary overview of the data and collection methods explaining the method of analysis and interpretation?
7. How is the data collected analysed and by whom? Is there enough capacity and capability? Why?
8. On a scale 1 to 5, where 1 means inadequate data collection while 5 means adequate data collection. How would you rate the data collection and storage by your unit? Why?
9. Is there any database that jointly receives rabies surveillance data from human and animal cases? If yes, is it used? How well does it work?
10. How do you think this compares to other parts of the surveillance system?
11. Are there data collected that are not used for any analysis or interpretation (redundancy)?
12. Can you describe what happens to the information generated from the analysis / how it is used? (any dissemination channels).
13. Is there anything else needed for effective rabies surveillance for which surveillance data are not currently collected and feasibly could be collected? Please describe.
14. What are the main challenges you and your colleagues face when it comes to data management within the surveillance system?

Timeliness:
Timeliness is defined as the time between any two defined steps within the surveillance system

1. Based on the map from part 1, What is the estimated time it takes for data about suspected rabies cases in humans/animal to be exchanged between the health and veterinary sectors?
   (Applies if there is actually of information)
2. How long does it take for data to be transferred/ reported from your facility (health/veterinary unit) to the health/veterinary database?
3. How long does it take data collected from health or veterinary facilities to be analysed by the surveillance officers?
4. On a scale 1 to 5 where 1 mean Excellent timeliness and 5 means poor timeliness: How good is the timeliness of the surveillance system to ensure rapid detection of suspected rabies cases for early case management? Please explain your reasoning. Also: what could be improved?

Usefulness/Impact of the surveillance system

A measure of the significance of the surveillance system in the control and prevention of rabies.

1. How are information or outputs generated from the surveillance data utilized? Is there room for improvement?

2. Has information generated by the surveillance system influenced the development of a control policy against rabies? If yes, can you describe it?
3. Is the information generated by the surveillance system used to contribute to the prioritization of rabies? If yes, can you describe it?
