# Supplementary material for: An assessment of the operationality and factors influencing the effectiveness of rabies surveillance in Gombe State, Nigeria
Source: PLoS Negl Trop Dis. 2024 May 7;18(5):e0012154. doi: 10.1371/journal.pntd.0012154 (PMC11108123; doi:10.1371/journal.pntd.0012154)
Supplement: S2 Text — (PDF) [file pntd.0012154.s002.pdf]

**Information and consent:**

Dear Sir/Madam, Good morning/afternoon and thank you for your time in taking part in this study.

My name is Adebanwo Kuye, I am a master's student from the Royal Veterinary College in London, United Kingdom, jointly supervised by Barbara Haesler from the Royal Veterinary College and Grace S.N Kia from the Ahmadu Bello University, Zaria.

I am investigating the surveillance system for dog bite cases and rabies in Gombe to understand the current operations of the system. In this interview I will ask you questions about six selected attributes of the surveillance system and information flow of the disease reporting in humans and animals.

Assessing the surveillance system and associated disease management can help to identify gaps and areas of strength in the system to increase the efficiency of the system. Evaluating rabies surveillance systems has been done in many countries which have enhanced their capacity towards rabies control.

It is entirely your choice to decide whether or not you wish to participate in this study. You can withdraw from this research if your decision to take part in this study changes. The decision to join this study will not cause you to lose any benefits. If you do feel uncomfortable with answering any of the questions kindly ask to move to the next question. The information you give will be treated confidentially, and your name will not be associated with anything you say. References to specific individuals will not be made in any oral or written presentations and your responses will be used only for research purposes.

I would be very grateful to get your support and collaboration in this research. The entire process should take approximately an hour to complete. Your participation is critical to the success of this study. However, it is entirely your choice to participate and your decision to be involved or not will not prevent you from getting access to the results generated from this research. The results produced from this study will not only benefit you but many other individuals in the state as well. Results obtained from this study will be used in writing a dissertation report and if feasible a peer-reviewed journal. Kindly note that this study has received ethical approval from the Royal Veterinary College's Review Board (Ref: URN SR2021-0147). I would be happy to communicate the results when the study is completed. You are free to ask any questions regarding this interview or the project in general if you want to. My Email address is: akuye20@rvc.ac.uk and my phone number is +2348185757700.

**CONSENT CERTIFICATE FOR STUDY PARTICIPANT**

ASSESSING THE SURVEILLANCE SYSTEM FOR DOG BITES AND RABIES IN GOMBE STATE, NORTH-EASTERN NIGERIA.

KUYE ADEBANWO ANUOLUWAPO (MSC ONE HEALTH)

Consent

By giving my consent, I confirm the following:

- I understand the description of this study and agree to take part as described in the project description.
- I am free to ask any question of the researcher and members of the project team in order to take part.
- I have read the information sheet about this study and I have asked questions about areas I needed clarity.
- I understand that it is entirely voluntary to participate in this research and I can withdraw at any time.
- This study may result in publication and I understand I will not be identified in any publication
- Any personally identifiable information will be kept separate from study data and no personal data will be released to any third party unless required to do so by law.
- No personal data from this study will be used in any way other than outlined in the project description.

**Do you give consent to participate in this study?** **Yes** ☐ **NO** ☐

**Do you give consent for this interview to be recorded? ?** **Yes** ☐ **NO** ☐

**Name:**

**Signature:**

**Date:**
