# Supplementary material for: An assessment of the operationality and factors influencing the effectiveness of rabies surveillance in Gombe State, Nigeria
Source: PLoS Negl Trop Dis. 2024 May 7;18(5):e0012154. doi: 10.1371/journal.pntd.0012154 (PMC11108123; doi:10.1371/journal.pntd.0012154)
Supplement: S3 Text — (PDF) [file pntd.0012154.s003.pdf]

MINISTRY OF AGRICULTURE AND ANIMAL HUSBANDRY
GOMBE STATE
AREA VETERINARY OFFICE
KASUWAN SHANU VETETRINARY CLINIC GOMBE

Date…………………………………

RE-DOG BITE REPORT

A. Victim (s)

i. Name……………………………………………………………………Age………………………Sex…………………………

ii. Address……

iii. Phone number…………………………………………………………Occupation…………………………………………

iv. Date of bite…………………………………………………………Time of bite……………………………………………

v. Date of report……

vi. Site of bite………

vii. Category/Frequency of bite…………………………………………………………………………………………

viii. Anti-Rabies vaccination pre-exposure prophylaxis status [Yes] or [No]

B. Dog Ownership

i. Ownership status……………Owned[ ] or Stray[ ]

ii. Name and address of Owner………………………………………………………………………………………

iii. G.S.M……
.

C. Culprit Dog

i. Name…………………………………………Color………………………Breed………………… Age………Sex……

ii. Anti-Rabies vaccination status: Up-to-date [ ] Never [ ] Unknown [ ] Expired [ ]

iii. Date of recent ARV given………………………………………………………………………………………………

iv. Source of the vaccine……………………………………………… Batch number……………………………………

D. Recommendation

………

………

………

………

Thank you

Veterinary Surgeon:
VCN No:
Phone No:
Signature/Stamp
